# Cerebral Erythropoietin Prevents Sex-Dependent Disruption of Respiratory Control Induced by Early Life Stress

**DOI:** 10.3389/fphys.2021.701344

**Published:** 2021-12-20

**Authors:** Elizabeth Elliot-Portal, Christian Arias-Reyes, Sofien Laouafa, Rose Tam, Richard Kinkead, Jorge Soliz

**Affiliations:** ^1^Centre de Recherche de l'Institut Universitaire de Cardiologie et de Pneumologie de Québec, Université Laval, Québec City, QC, Canada; ^2^High Altitude Pulmonary and Pathology Institute (HAPPI–IPPA), La Paz, Bolivia

**Keywords:** sleep apnea, corticosterone, chemosensing, neuroprotection, brainstem

## Abstract

Injuries that occur early in life are often at the root of adult illness. Neonatal maternal separation (NMS) is a form of early life stress that has persistent and sex-specific effects on the development of neural networks, including those that regulate breathing. The release of stress hormones during a critical period of development contributes to the deleterious consequences of NMS, but the role of increased corticosterone (CORT) in NMS-induced respiratory disturbance is unknown. Because erythropoietin (EPO) is a potent neuroprotectant that prevents conditions associated with hyperactivation of the stress neuroaxis in a sex-specific manner, we hypothesized that EPO reduces the sex-specific alteration of respiratory regulation induced by NMS in adult mice. Animals were either raised under standard conditions (controls) or exposed to NMS 3 h/day from postnatal days 3–12. We tested the efficacy of EPO in preventing the effects of NMS by comparing wild-type mice with transgenic mice that overexpress EPO only in the brain (Tg21). In 7-days-old pups, NMS augmented CORT levels ~2.5-fold by comparison with controls but only in males; this response was reduced in Tg21 mice. Respiratory function was assessed using whole-body plethysmography. Apneas were detected during sleep; the responsiveness to stimuli was measured by exposing mice to hypoxia (10% O_2_; 15 min) and hypercapnia (5% CO_2_; 10 min). In wild-type, NMS increased the number of apneas and the hypercapnic ventilatory response (HcVR) only in males; with no effect on Tg21. In wild-type males, the incidence of apneas was positively correlated with HcVR and inversely related to the tachypneic response to hypoxia. We conclude that neural EPO reduces early life stress-induced respiratory disturbances observed in males.

## Introduction

There is now a strong scientific consensus acknowledging that stress experienced chronically or during a critical period of development is a major cause of adult disease (Shonkoff et al., 2009; Shonkoff, 2016; Kivimäki and Steptoe, 2018; Nelson and Gabard-Durnam, 2020). Stress has persistent and sex-specific effects on health and the brain is a major target (McEwen et al., 2015). One neural system of considerable interest is the neural system regulating breathing because pathology in this homeostatic control system underlies important clinical disorders, such as sleep apnea (SA; Dempsey et al., 2010; White and Younes, 2012; Eckert, 2018). Early life stress comes in many forms, and basic and clinical studies consistently show that adverse conditions, such as low socioeconomic status or poor parental care, can compromise the respiratory control system at various life stages (Genest et al., 2004; Spilsbury et al., 2006; Kinney and Thach, 2009; Boss et al., 2011; D’Amato et al., 2011; Fournier et al., 2013; Battaglia et al., 2014; Guglielmi et al., 2019; Tenorio-Lopes and Kinkead, 2021). In humans, these stressors are often associated with significant confounding factors related to maternal health and lifestyle thus compromising our ability to conclude on the link between stress and respiratory control dysfunction. Animal studies offer better control over those limiting factors and neonatal maternal separation (NMS) is a well-established and clinically relevant form of stress commonly used in basic research. Inadequate maternal care poses no direct threat to respiratory homeostasis but disruption of the balance between excitatory and inhibitory mechanisms regulating respiratory control contributes to the impacts of NMS on those systems (Genest et al., 2007; Gulemetova et al., 2013; Baldy et al., 2017). NMS also disrupts the function of the stress pathways by affecting the paraventricular nucleus of the hypothalamus (PVH; Liu et al., 1997; Genest et al., 2007; Ulrich-Lai and Herman, 2009; Gulemetova et al., 2013). Owing to its direct projections into structures generating the respiratory rhythm, integrating respiratory stimuli, and motoneurons controlling the diaphragm and upper airways, the PVH exerts a “top down” influence on respiratory function (Tenorio-Lopes and Kinkead, 2021).

Dysregulation of the hypothalamo-pituitary-adrenal (HPA) axis results in an excessive neuroendocrine response to challenges and the prolonged elevation of corticosterone (CORT) associated with this condition is sufficient to promote oxidative stress (Spiers et al., 2015; Rousseau et al., 2017). In adult male (but not females) rats, NMS augments basal activity of the HPA axis, mean arterial blood pressure, and the hypoxic ventilatory response (HVR); NMS also increases respiratory instability during sleep (Genest et al., 2004, 2007; Kinkead et al., 2005, 2008). While the disruption of the stress pathways may contribute directly and indirectly to anomalies in respiratory control observed in male rats, the neonatal origins of this problem are unknown. Corticosterone is one of the main stress hormones; because it acts *via* nuclear receptors, CORT acts as a transcription factor and can induce epigenetic modifications (Darnaudery and Maccari, 2008; Lightman, 2008; Nishi et al., 2014). As a result, exposure to stress hormones during a critical period of development has emerged as a significant contributor to the deleterious consequences of early life stress on the brain (Hamada and Matthews, 2019); however, its role in NMS-induced respiratory disturbance is unknown.

Erythropoietin (EPO) is a promising neuroprotective agent with widespread clinical relevance (Grasso et al., 2007; Soliz et al., 2020). EPO mediates neuroprotection after traumatic injury, hypoxia-ischemic injuries, excitotoxicity, and inflammatory brain injury (Brines and Cerami, 2005). At neonatal ages, EPO reduces perinatal brain injury (Rangarajan and Juul, 2014; Juul and Pet, 2015) and improves neurological outcomes in very prematurely born neonates (McPherson and Juul, 2008; Juul et al., 2015; Juul and Pet, 2015). Moreover, EPO has major effects in the regulation of metabolic homeostasis by modulating the activity of the HPA axis (Tringali et al., 2007). EPO attenuates the neuroendocrine response under conditions of chronic stress or more acute ischemic–hypoxic brain challenges (Dey et al., 2020). In fact, in a rat hypothalamic explant model, corticotropin-releasing hormone (CHR) was significantly reduced by EPO in male animals only (Tringali et al., 2007; Dey et al., 2020). Taking advantage of this unique property of EPO, we used Tg21 mice (that constitutively overexpress human EPO solely in the brain) to test the hypothesis that EPO prevents the persistent and sex-specific disruptions of respiratory control resulting from NMS. Our results show that EPO prevents the increase of CORT secretion at P7 and frequency of apnea in adulthood (at P60) induced by NMS in male mice.

## Materials and Methods

### Animals

All experiments were carried out using wild-type (WT) and heterozygous transgenic (Tg21) mice. Tg21 mice show constitutive overexpression of EPO in the brain only (Ruschitzka et al., 2000; Wiessner et al., 2001). WT animals were obtained after crossing six generations of heterozygous Tg21 mice with C57BL6 animals. All mice were bred and housed under standard conditions in the IUCPQ animal facilities. The animals were kept on a 12:12 light:dark cycle, with food and water provided *ad libitum*. The animal experiments were approved by the Animal Protection Committee of Laval University, QC, Canada.

### Mating Procedures and NMS Protocol

**Figure 1:**
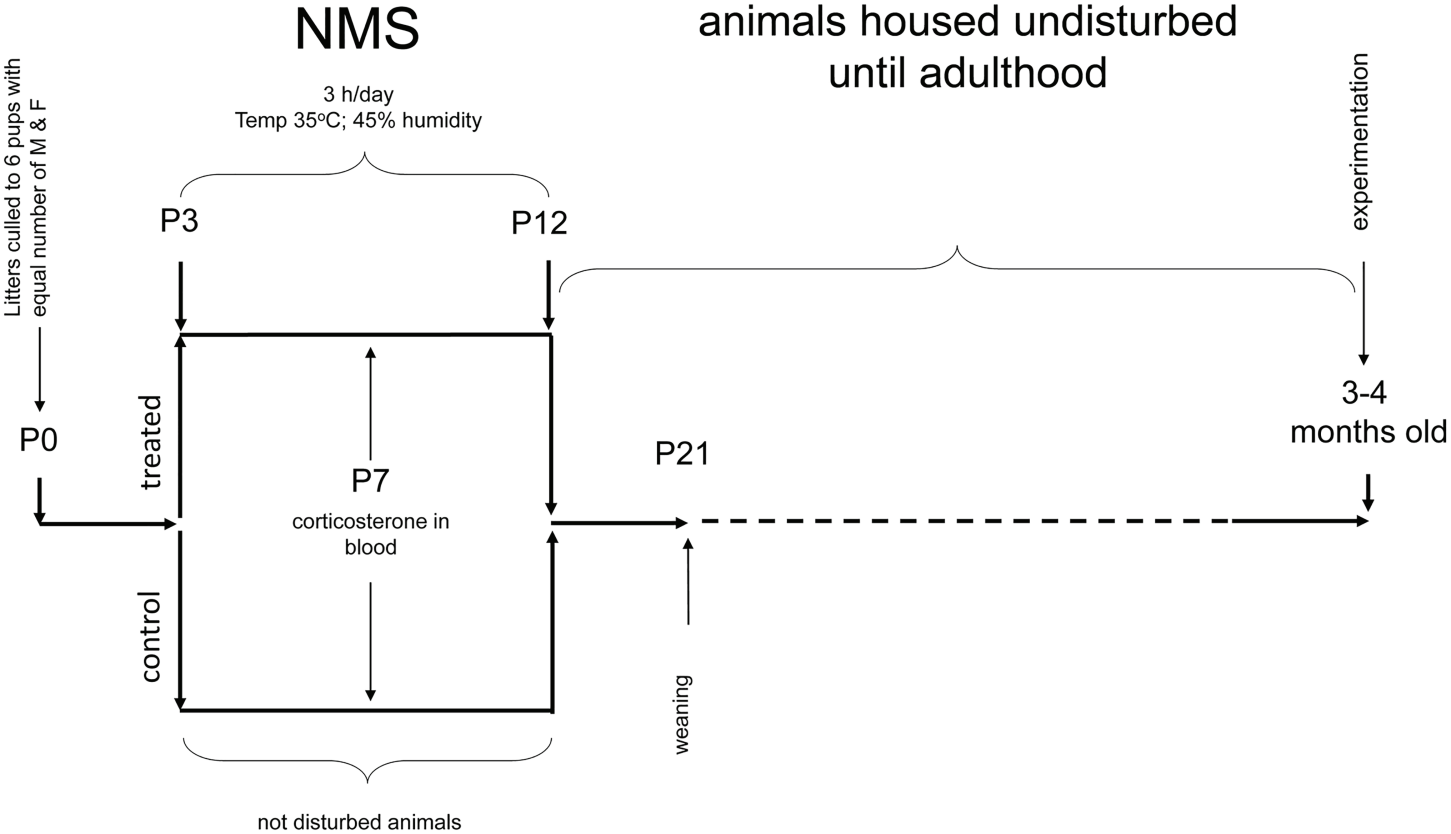
Schematic representation of the neonatal maternal separation (NMS) protocol and evaluation of respiratory parameters in adults. WT and Tg21 animals were exposed to NMS between postnatal ages P3 and P12. Undisturbed animals during P3-P12 were used as control groups. Plasma corticosterone (CORT) levels were evaluated at P7 in a separate series of experiments. Resting breathing and in response to ventilatory stimuli were assessed when the animals reached adulthood (3–4 months old).

Mice pups were obtained by crossing WT (virgin) females with Tg21 males. On the 2nd day of birth, the litters were reduced to a maximum of six pups, with the same number of males and females, when possible. Between postnatal ages P3 and P12 (10 days), the pups were exposed to NMS (males WT: *n* = 9 and Tg21: *n* = 7; females WT: *n* = 6 and Tg21: *n* = 6), which consists of isolating the offspring from their mother 3 h/day (from 9:00am at 12:00pm) from postnatal day 3–12. Pups are placed in an incubator where the temperature and humidity are kept constant (35°C and 45% RH, respectively). Pups are isolated from each other by acrylic (Genest et al., 2007; Fournier et al., 2015; Tenorio-Lopes et al., 2020). As discussed elsewhere (Lehmann and Feldon, 2000; Gulemetova and Kinkead, 2011), animals that are undisturbed during that period and remain in their nest are the best control groups for this type of research (males WT: *n* = 9 and Tg21: *n* = 7; females WT: *n* = 6 and Tg21: *n* = 8). The pups were weaned at 21 days of age and reared under the standard conditions. All the experiments for the evaluation of apneic events and recording of ventilation were carried out in adulthood, between 3 and 4 months of age (Figure 1).

### Corticosterone Assay

In a separate series of experiments (WT: males *n* = 6; females *n* = 7; Tg21: males *n* = 8; females *n* = 6), we determined whether an increase in cerebral EPO attenuates the CORT release normally observed in pups during the NMS protocol (Gulemetova and Kinkead, 2011). Terminal blood samples were collected at postnatal day 7 under baseline conditions from 17 NMS mice and controls from both genotypes (Tg21 and WT). Mice were deeply anesthetized, and blood was collected from the left ventricle. The sample was transferred to a vacutainer tube containing EDTA (Becton Dickinson, Québec, Canada). Centrifugation for 15 min at 2,000 x *g* was used to separate the plasma. Plasma samples were stored at −20°C. Corticosterone levels were measured with a microplate spectrophotometer (μ-Quant, Bio-Tek Instruments Inc., Winooski, VT, United States) and a standard curve (linearized by a log–log transformation) was used to calculate CORT concentrations.

### Respiratory Recordings

A whole-body plethysmograph for mice (Emka Technologies, Paris, France) was used to record the ventilatory parameters. As described elsewhere (Ballot et al., 2015), this non-invasive technique allows non-sedated animals to move freely and within the plethysmography chamber. Assessment of respiratory measurements started when the animal in the chamber appeared calm and the respiratory signal was devoid of movement artefacts. The airflow inside the plethysmography chamber (generated by the respiratory movements of the animals) was transformed into electrical signals and recorded using Spike 2 software (Cambridge Electronic Design, Cambridge, United Kingdom). These recordings allowed the calculation of respiratory rate (*f*_r_), tidal volume (V_t_), and minute ventilation (V_e_ = f_r_ x V_t_). Tidal volumes were expressed in ml of BTPS by correcting the raw V_t_ data with the barometric pressure, humidity, chamber temperature, and body temperature (thermocouple probe – Physitemp Instruments, Clifton NJ, United Kingdom; Drorbough and Fenn, 1955; Bartlett and Tenney, 1970). The corrected V_t_ was normalized to body weight (ml BTPS/100 g).

### Breathing at Rest in Response to Ventilatory Stimuli

These experiments were performed when the animals reached adulthood (3–4 months old – Figure 1). Once inside the plethysmograph chamber, it takes the animals about an hour to become familiar with their surroundings and become calm. Once in this state, the respiratory signal is recorded for 2 h under ambient air conditions (normoxia). This wide window of respiratory records, which contains stable periods of sleep (non-REM), allows the evaluation of apneic events. Determination of sleep/wake states was based on the variability of the respiratory signal as described recently (Bastianini et al., 2017). Comparing of the identification of sleep/wake states with this non-invasive method with the classical approach involving EEG and EMG monitoring shows that the two methods agree 90% of the time (Bastianini et al., 2017). A non-invasive approach obviates concerns related to confounding effects of the stress associated with surgical implantation of EEG–EMG electrodes. An interruption of the signal was considered an apneic event when breathing stopped for a length of two or more regular breaths (Montandon et al., 2006). Next, the respiratory signal was recorded under conditions of hypoxia (10% O_2_; 15 min) and hypercapnia (21% O_2_, 5% CO_2_, in N_2_; 10 min), separated by a brief return to normoxia (10 min).

### Data Analyses and Statistics

Corticosterone concentration, number of apneas (total, spontaneous, and post-sigh), sighs per hour, and ventilatory parameters (V_E_, *f*_R_, and V_T_) were analyzed using three-way ANOVA analyses. Sex (male or female), strain (WT or Tg21), and treatment (control or NMS) were defined as factors. Ventilatory parameters during normoxia (Nx), hypoxia (Hx), and hypercapnia (Hc) were analyzed separately. The effect of the exposure to Nx, Hx, and Hc on ventilatory parameters was assessed by repeated measures three-way ANOVA tests separately for males and females using exposure (repeated measures), strain, and treatment as factors. Ventilatory responses to hypoxia (HVR) and hypercapnia (HcVR) were calculated as the difference of hypoxic and hypercapnic ventilation minus baseline ventilation, respectively. The relationships between apneas and the ventilatory response to hypercapnia, and between apneas and the respiration frequency during hypoxia were assessed by Pearson’s correlation analyses. The percent change in *f*_R_ was calculated as: [*f*_R(Hx)_ – (*f*_R(baseline)_)*100]/*f*_R(baseline)_. For all analyses, when significant differences were found, uncorrected Fisher’s LSD *post hoc* tests were performed. Results from *post hoc* tests are reported in the figures using symbols. Table 1 contains a detailed description of the value of *p* found for the ventilatory parameters as a function of sex, animal strain, and treatment. All analyses and graphs were done with the GraphPad prism 9.1 software (La Jolla, CA, United States). The data are shown as means ± SD. Differences were considered significant at *p* < 0.05.

**Table 1:** Three-way ANOVA results for the effect of sex (males vs. females), strain (WT vs. Tg21), and treatment (control vs. NMS) on the concentration of CORT; number of total, spontaneous, and post-sigh apneas; number of sighs; and minute ventilation (V*_E_*), respiratory frequency (*f*_R_), and tidal volume (V_T_) during Nx, Hx, Hc, HVR, and HcVR.

	Sex effect		Strain effect		Treatment effect		Sex x strain		Sex x treatment		Strain x treatment		Sex x strain x treatment	
	*F*_(d.f.1, d.f.2)_	*p*	*F*_(d.f.1, d.f.2)_	*p*	*F*_(d.f.1, d.f.2)_	*p*	*F*_(d.f.1, d.f.2)_	*p*	*F*_(d.f.1, d.f.2)_	*p*	*F*_(d.f.1, d.f.2)_	*p*	*F*_(d.f.1, d.f.2)_	*p*
Corticosterone	_(1, 40)_ = 43.48	<0.001	_(1, 40)_ = 16.16	=0.0003	_(1, 40)_ = 39.58	<0.001	_(1, 40)_ = 12.92	=0.0009	_(1, 40)_ = 29.10	<0.001	_(1, 40)_ = 10.23	=0.003	_(1, 40)_ = 6.909	=0.012
Apneas														
Total	_(1, 43)_ = 5.10	=0.029	_(1, 43)_ = 1.40	=0.244	_(1, 43)_ = 6.42	=0.015	_(1, 43)_ = 2.04	=0.160	_(1, 43)_ = 6.01	=0.018	_(1, 43)_ = 0.770	=0.385	_(1, 43)_ = 9.70	=0.003
Spontaneous	_(1, 43)_ = 0.226	=0.637	_(1, 43)_ = 0.190	=0.665	_(1, 43)_ = 3.35	=0.074	_(1, 43)_ = 4.21	=0.046	_(1, 43)_ = 2.02	=0.162	_(1, 43)_ = 0.965	=0.331	_(1, 43)_ = 1.94	=0.171
Post-sigh	_(1, 42)_ = 0.261	=0.612	_(1, 42)_ = 5.94	=0.019	_(1, 42)_ = 12.5	=0.001	_(1, 42)_ = 2.78	=0.103	_(1, 42)_ = 4.78	=0.035	_(1, 42)_ = 6.92	=0.012	_(1, 42)_ = 0.803	=0.376
Sighs	_(1, 43)_ = 8.789	=0.005	_(1, 43)_ = 0.001	=0.922	_(1, 43)_ = 4.416	=0.041	_(1, 43)_ = 1.158	=0.288	_(1, 43)_ = 5.229	=0.027	_(1, 43)_ = 5.930	=0.019	_(1, 43)_ = 0.074	=0.786
V*_E_*														
Nx	_(1, 41)_ = 13.4	<0.001	_(1, 41)_ = 0.01	=0.923	_(1, 41)_ = 0.167	=0.685	_(1, 41)_ = 0.002	=0.965	_(1, 41)_ = 0.273	=0.604	_(1, 41)_ = 4.74	=0.035	_(1, 41)_ = 1.23	=0.274
Hx	_(1, 41)_ = 2.09	=0.156	_(1, 41)_ = 2.64	=0.112	_(1, 41)_ = 2.34	=0.134	_(1, 41)_ = 15.1	<0.001	_(1, 41)_ = 0.137	=0.714	_(1, 41)_ = 2.09	=0.156	_(1, 41)_ = 3.62	=0.064
Hc	_(1, 43)_ = 94.7	<0.001	_(1, 43)_ = 76.3	<0.001	_(1, 43)_ = 22.2	<0.001	_(1, 43)_ = 4.60	=0.038	_(1, 43)_ = 0.890	=0.351	_(1, 43)_ = 41.4	<0.001	_(1, 43)_ = 8.10	=0.007
HVR	_(1, 39)_ = 2.33	=0.135	_(1, 39)_ = 3.30	=0.077	_(1, 39)_ = 2.55	=0.118	_(1, 39)_ = 12.1	=0.001	_(1, 39)_ = 0.0007	=0.978	_(1, 39)_ = 0.15	=0.697	_(1, 39)_ = 1.02	=0.319
HcVR	_(1, 42)_ = 57.5	<0.001	_(1, 42)_ = 84.5	<0.001	_(1, 42)_ = 28.3	<0.001	_(1, 42)_ = 3.86	=0.056	_(1, 42)_ = 1.11	=0.298	_(1, 42)_ = 27.8	<0.001	_(1, 42)_ = 12.5	=0.001
*f*_R_														
Nx	_(1, 43)_ = 9.15	=0.004	_(1, 43)_ = 4.84	=0.033	_(1, 43)_ = 0.099	=0.754	_(1, 43)_ = 2.48	=0.122	_(1, 43)_ = 0.043	=0.836	_(1, 43)_ = 8.49	=0.006	_(1, 43)_ = 0.20	=0.653
Hx	_(1, 42)_ = 9.21	=0.004	_(1, 42)_ = 8.07	=0.007	_(1, 42)_ = 4.61	=0.038	_(1, 42)_ = 3.95	=0.053	_(1, 42)_ = 16.5	<0.001	_(1, 42)_ = 0.0002	=0.990	_(1, 42)_ = 2.93	=0.094
Hc	_(1, 42)_ = 1.39	=0.245	_(1, 42)_ = 1.06	=0.309	_(1, 42)_ = 0.091	=0.765	_(1, 42)_ = 0.0005	=0.982	_(1, 42)_ = 0.252	=0.619	_(1, 42)_ = 0.09	=0.768	_(1, 42)_ = 0.64	=0.427
HVR	_(1, 42)_ = 89.4	<0.001	_(1, 42)_ = 40.0	<0.001	_(1, 42)_ = 3.84	=0.057	_(1, 42)_ = 121	<0.001	_(1, 42)_ = 18.1	<0.001	_(1, 42)_ = 2.61	=0.114	_(1, 42)_ = 2.34	=0.134
HcVR	_(1, 42)_ = 54.3	<0.001	_(1, 42)_ = 21.5	<0.001	_(1, 42)_ = 0.211	=0.648	_(1, 42)_ = 16.2	<0.001	_(1, 42)_ = 0.408	=0.526	_(1, 42)_ = 0.01	=0.912	_(1, 42)_ = 3.75	=0.059
V_T_														
Nx	_(1, 40)_ = 13.78	=0.0006	_(1, 40)_ = 0.6025	=0.442	_(1, 40)_ = 0.076	=0.783	_(1, 40)_ = 0.08	=0.780	_(1, 40)_ = 0.073	=0.788	_(1, 40)_ = 1.21	=0.277	_(1, 40)_ = 0.22	=0.642
Hx	_(1, 39)_ = 1.821	=0.185	_(1, 39)_ = 0.5620	=0.458	_(1, 39)_ = 10.51	=0.002	_(1, 39)_ = 3.96	=0.053	_(1, 39)_ = 0.004	=0.946	_(1, 39)_ = 0.24	=0.626	_(1, 39)_ = 0.11	=0.739
Hc	_(1, 35)_ = 0.03	=0.871	_(1, 35)_ = 0.0026	=0.959	_(1, 35)_ = 0.047	=0.829	_(1, 35)_ = 1.265	=0.268	_(1, 35)_ = 0.993	=0.326	_(1, 35)_ = 4.949	=0.032	_(1, 35)_ = 7.549	=0.009
HVR	_(1, 43)_ = 4.119	0.0486	_(1, 43)_ = 0.0899	=0.765	_(1, 43)_ = 13.62	=0.0006	_(1, 43)_ = 0.48	=0.492	_(1, 43)_ = 3.154	=0.082	_(1, 43)_ = 0.417	=0.522	_(1, 43)_ = 0.235	=0.630
HcVR	_(1, 43)_ = 1.66	=0.204	_(1, 43)_ = 0.0203	=0.887	_(1, 43)_ = 4.13	=0.048	_(1, 43)_ = 0.169	=0.683	_(1, 43)_ = 0.212	=0.646	_(1, 43)_ = 2.661	=0.110	_(1, 43)_ = 3.637	=0.063

## Results

### NMS Induces Sex-Specific Stimulation of Corticosterone Release in Wild-Type, but Not Tg21 Pups

**Figure 2:**
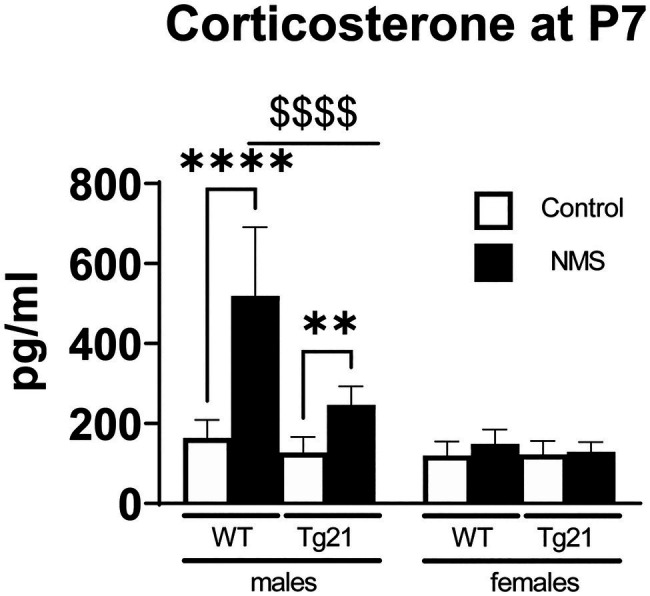
Plasma concentration of CORT was evaluated at postnatal day 7 (P7) in WT (males *n* = 6; females *n* = 7) and Tg21 (males *n* = 8; females *n* = 6) mice exposed (or not – control) to NMS. Data reported as means ± SD. * Indicates differences between treatments (controls vs. NMS). $ Indicates differences between strains (WT vs. Tg21). **: *p* < 0.01; ****, or $$$$: *p* < 0.0001.

In wild-type, evaluation of plasma CORT levels in 7-day-old mice (P7) shows that the NMS protocol stimulated the HPA axis in males (not females); this effect was not observed in Tg21 (Table 1; Figure 2). By the end of the protocol (P12), the body weight of pups subjected to NMS was lower than controls (Tables 1 and 2); this effect was not sex-specific or influenced by the pup’s genotype (Tables 1 and 2). At adulthood, males generally weighted more than females. In males, NMS augmented the body weight regardless of the genotype. In females, body weights of wild-type were greater in NMS than controls; NMS had an opposite effect in Tg21 mice (Tables 1 and 2). Basal body temperature of adult females was higher than males (Table 2).

**Table 2:** Body weight of animals at postnatal and adult ages.

		WT		Tg21	
		Control (*n* = 9)	NMS (*n* = 11)	Control (*n* = 10)	NMS (*n* = 10)
Body weight pups at P12 (g)	Male	5.9 ± 0.5	5.4 ± 0.4	5.8 ± 0.3	5.6 ± 0.7
	Female	5.9 ± 0.5	5.3 ± 0.3^**^	5.9 ± 0.4	5.5 ± 0.4
Body weight adults (g)	Male	30.4 ± 0.5	31.2 ± 0.6	29.0 ± 1.0	31.1 ± 0.6^***^
	Female	24.8 ± 0.2^####^	25.1 ± 0.7^***^^,^^####^	24.1 ± 0.6^####^	23.5 ± 0.4^####^
Body temp adults (°C)	Male	33.8 ± 0.4	33.5 ± 0.2^*^	33.1 ± 0.1	33.1 ± 0.2^***^
	Female	34.5 ± 0.2^##^	35.2 ± 0.8	34.5 ± 0.2^####^	34.1 ± 0.3

*Indicates differences between treatments (controls vs. NMS). # Indicates differences between sexes (male vs. female).

* *p < 0.05*;

** *p < 0.01*,

## *p < 0.01*;

*** *p < 0.001*;

^###^ *p < 0.001; and*

#### *p < 0.0001*.

### Neural EPO Prevents NMS-Related Increase in the Number of Apneic Events in Males

**Figure 3:**
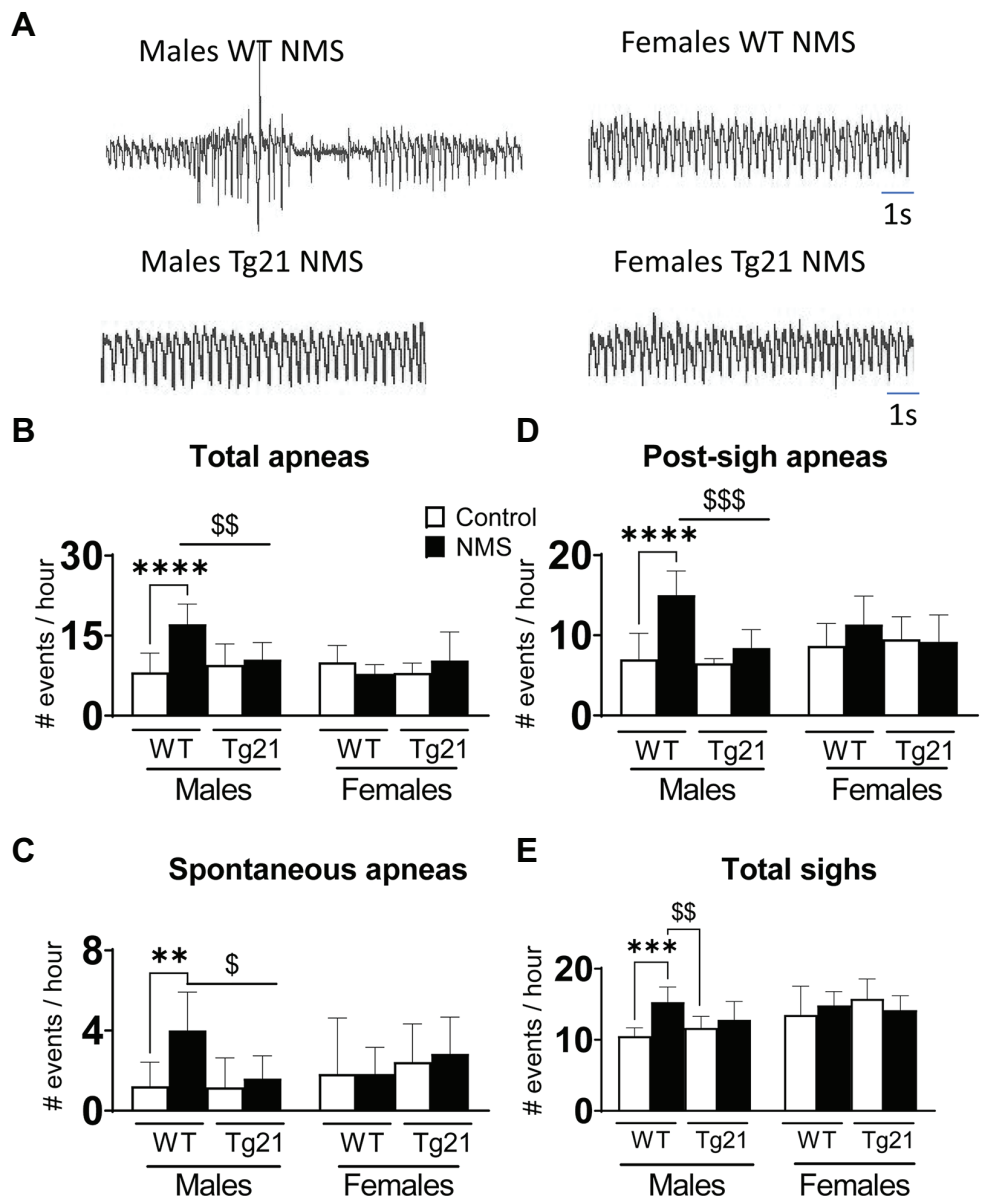
Number of apneic (total, spontaneous, and post-sigh) and sighs events in male (WT: control *n* = 9; NMS *n* = 9; Tg21: control *n* = 7; NMS *n* = 7) and female (WT: control *n* = 6; NMS *n* = 6; Tg21: control *n* = 8; NMS *n* = 6) mice previously exposed (or not – control) to NMS. **(A)** Original recordings comparing resting respiration records between WT and Tg21 male and female animals exposed to NMS. The number of total **(B)**, spontaneous **(C)**, and post-sight apneas **(D)** and total sight **(E)** were significantly increased in NMS-exposed male WT mice. No effect of NMS was observed in male Tg21 mice, neither in WT or Tg21 female mice exposed, or not, to NMS. Data reported as means ± SD. * Indicates differences between treatments (controls vs. NMS). $ Indicates differences between strains (WT vs. Tg21). $: *p* < 0.05; **, or $$: *p* < 0.01; ***, or $$$: *p* < 0.001; and ****: *p* < 0.0001.

In WT males, the apnea frequency of adult mice subjected to NMS was more than two times greater than that of controls. This difference was not observed in Tg21 mice, regardless of their sex (Figures 3A,B; Table 1). This effect was due to an increase in post-sigh and spontaneous apneas (Figures 3C,D; Table 1). Sighs were significantly increased in WT NMS males (Figure 3E; Table 1).

### NMS Increases the Hypercapnic Ventilation in WT, But Not in Tg21 Male Animals

**Figure 4:**
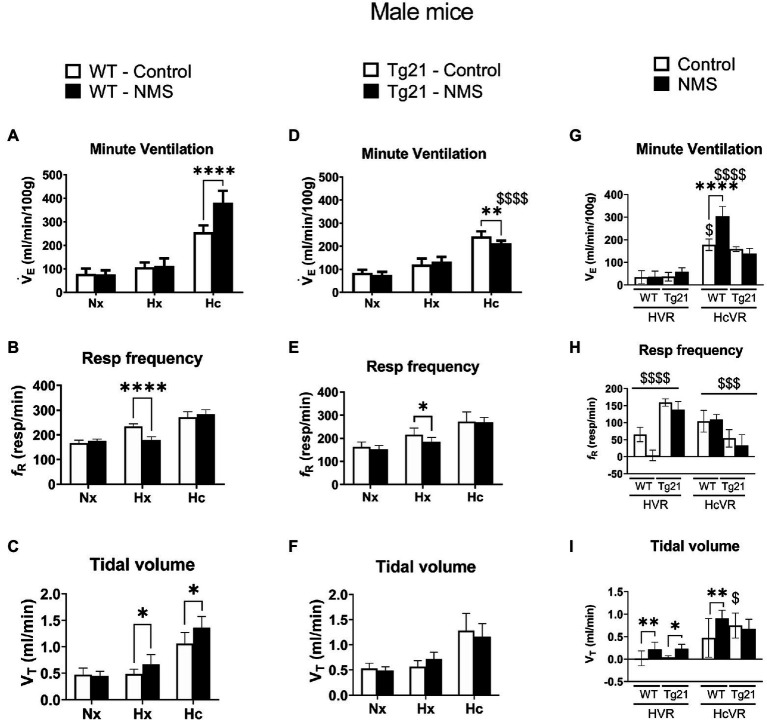
**(A)** Minute ventilation (V*_E_*), **(B)** respiratory frequency (*f*_R_), and **(C)** tidal volume (V_T_) evaluated under conditions of normoxia (Nx), hypoxia (Hx), and hypercapnia (Hc) in male WT mice that were either raised under control condition or subjected to NMS (control *n* = 9; NMS *n* = 7). **(D–F)** report the same measurements obtained in male Tg21 mice (control *n* = 6; NMS *n* = 6). **(G)** Minute ventilation, **(H)** respiratory frequency, and **(I)** tidal volume under hypoxic (hypoxic ventilatory response – HVR = Hx – Nx) and hypercapnic (hypercapnic ventilatory response – HcVR = Hc – Nx) stimulation show that NMS does not affect minute ventilation in HVR in any strain, but significantly increases HcVR only in WT mice. Data reported as means ± SD. * Indicates differences between treatments (controls vs. NMS). $ Indicates differences between strains (WT vs. Tg21). *, $: *p* < 0.05; **: *p* < 0.01; $$$: *p* < 0.001; and ****, $$$$: *p* < 0.0001.

**Figure 5:**
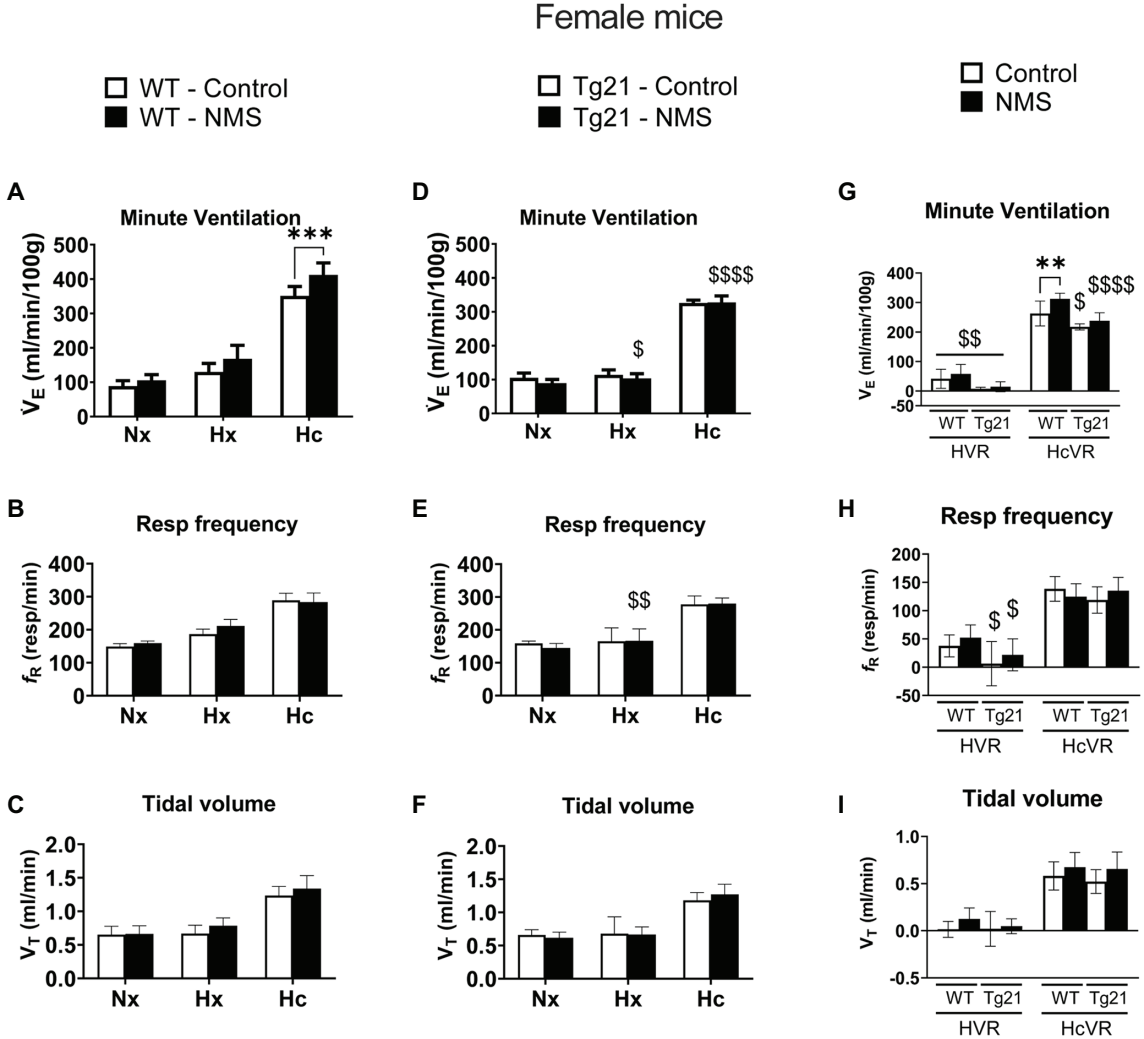
**(A)** Minute ventilation (V*_E_*), **(B)** respiratory frequency (*f*_R_), and **(C)** tidal volume (V_T_) evaluated under conditions of Nx, Hx, and Hc in female WT mice that were either raised under control condition or subjected to NMS (control *n* = 10; NMS *n* = 7). **(D–F)** report the same measurements obtained in male Tg21 mice (control *n* = 5; NMS *n* = 6). **(G)** Minute ventilation, **(H)** respiratory frequency, and **(I)** tidal volume under hypoxic (hypoxic ventilatory response – HVR = Hx – Nx) and hypercapnic (hypercapnic ventilatory response – HcVR = Hc – Nx) stimulation show that NMS does not affect minute ventilation in HVR in any strain, but significantly increases HcVR only in WT mice. Data reported as means ± SD. * Indicates differences between treatments (controls vs. NMS). $ Indicates differences between strains (WT vs. Tg21). $: *p* < 0.05; **, $$: *p* < 0.01; ****: *p* < 0.001; and $$$$: *p* < 0.0001.

We first compared ventilatory activity at rest (normoxia; Nx) and data show that although NMS treatment and the mouse’s genotype influenced breathing pattern (breathing frequency vs. tidal volume), this had no net effect on minute ventilation (Figures 4, 5; Table 1). Exposure to hypoxia augmented ventilatory activity (Three-way RM ANOVA Males: hypoxia effect: *F*_(2, 48)_ = 566.1 *p* < 0.0001; hypoxia_x_Strain_x_Treatment *F*_(2, 48)_ = 26.41 *p* < 0.0001; Figures 4G, 5G) and expressing the minute ventilation response as the difference of the stimuli minus the baseline showed that overexpression of neural EPO attenuated the HVR in females but not males (Three-way RM ANOVA: Sex_x_Strain: *F*_(1, 39)_ = 12.1 *p* = 0.001); this effect was mainly due to a reduced frequency response. NMS did not affect the HVR significantly (Figures 4G, 5G). Conversely, NMS augmented the HcVR in wild-type males (Figure 4G) and females (Figure 5G); this effect mainly reflects an augmented tidal volume response in males. The HcVR of Tg21 was unaffected by NMS (Fisher’s LSD; Tg21-control vs. Tg21-NMS *p*_males_ = 0.229; *p*_females_ = 0.264; Figures 4G, 5G).

### Anomalies in Respiratory Reflexes Correlate With NMS-Related Rise in Apnea Frequency in Males

**Figure 6:**
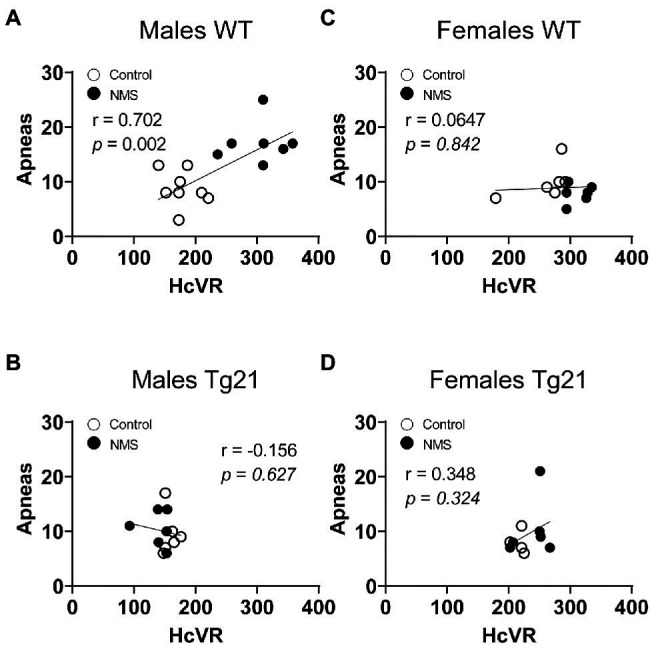
Correlation between apneic events and hypercapnic ventilatory response (HcVR = Hc – Nx) in WT and Tg21 male **(A,B)** and female **(C,D)** mice previously exposed (or not – control) to NMS.

**Figure 7:**
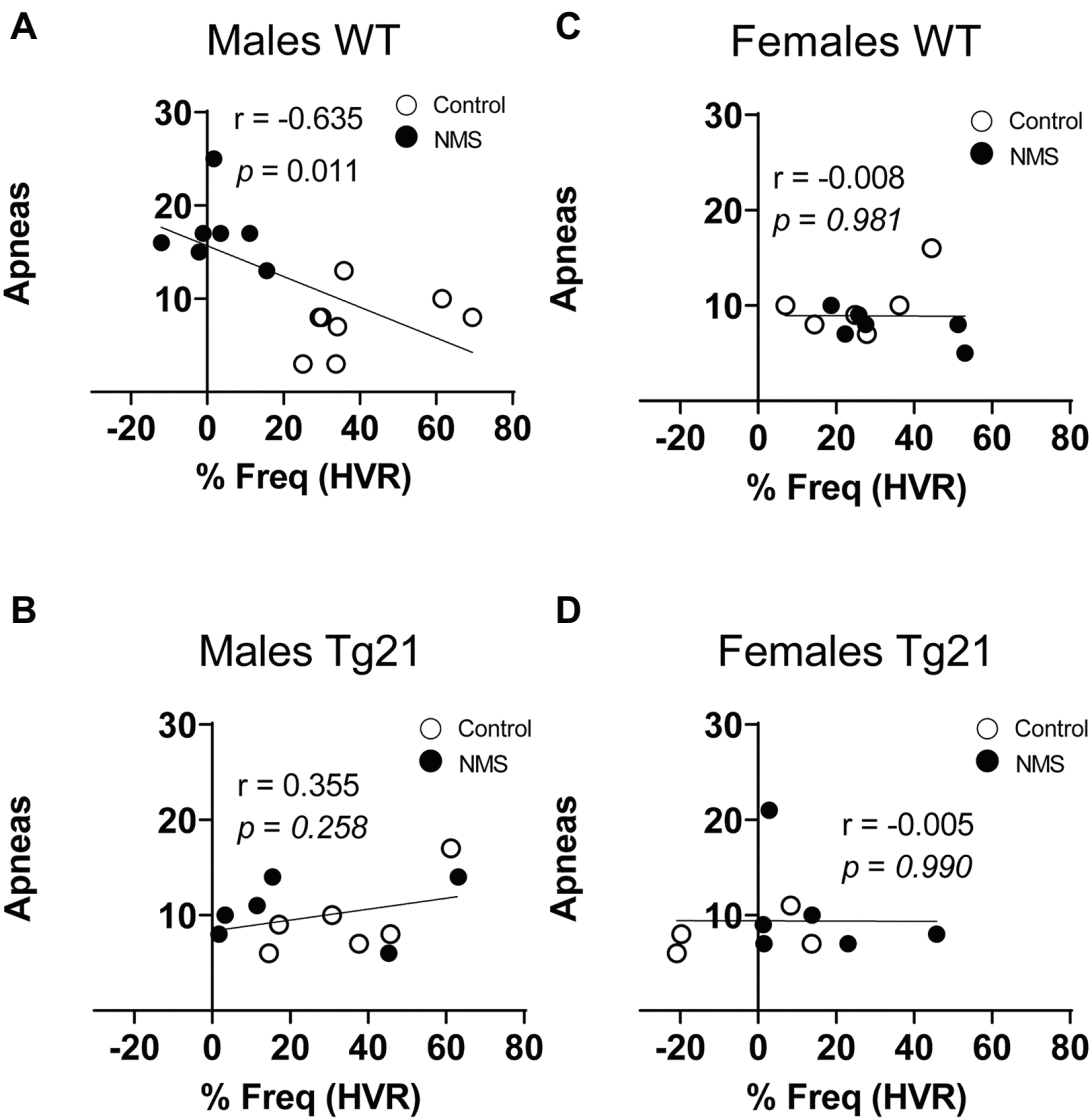
Correlation between apneic events and percentage of change in respiratory frequency (in HVR) in WT and Tg21 male **(A,B)** and female **(C,D)** mice previously exposed (or not – control) to NMS.

Because abnormal chemoreflexes are an important mechanism in the pathophysiology of sleep apnea (Dempsey et al., 2010; White and Younes, 2012; Eckert, 2018), we performed a correlation matrix to evaluate the relationships between the intensity of the HVR and HcVR (and tidal volume and frequency components) and the frequency of apneic events recorded during non-REM sleep (Figures 6, 7). Significant relationships were observed only in wild-type males; in this group, NMS mice displayed the largest apnea frequency. In these animals, the apnea frequency was directly proportional to the HcVR (Figure 6A) but was inversely related to the rise in breathing frequency measured during hypoxia (Figure 7A).

## Discussion

There is growing evidence indicating that early life stress has long-lasting and sex-specific consequences on respiratory control (Tenorio-Lopes and Kinkead, 2021); however, nearly all the experimental data supporting this concept originate from rats and the proximal (neonatal) mechanisms leading to an abnormal programming of the respiratory phenotype are unknown. In wild-type mice, confirmation that NMS augments CORT in male (but not female) pups (P7) and leads to sex-specific respiratory control dysfunction in adult males are important because they further establish the link between neonatal stress and the emergence of respiratory control disorders later in life. From a practical perspective, these data validate the use of transgenic models that this species has to offer. Building on those basic observations, our demonstration that in males, neural EPO attenuates both the rise in CORT in male pups and respiratory control dysfunction in adults supports our main hypothesis and suggests that elevation of circulating CORT during a critical period of development contributes to stress-related respiratory control dysfunction later in life.

### Neonatal Stress and Sex-Specific Activation of the Stress Response in Newborn

In rat pups, exposure to most types of stressors during the first two postnatal weeks elicits a weak adrenal response (Vázquez, 1998). This stress-hyporesponsive period protects the organism from the deleterious effects of various neonatal stressors, both physical and psychological, on growth and development (Wood and Walker, 2015). The stimuli that the mother provides to her pups during the postnatal period attenuates the function of the stress axis and thus contribute to the HPA’s hypo-responsiveness (Suchecki, 2018). Consequently, depriving the pups from maternal care leads to a robust increase in CORT as we have reported here. Sex-based differences in HPA axis function are well documented in sexually mature animals, but the fact that in wild-type mice, NMS augmented CORT only in P7 males indicates that this dimorphism is present during early development. This observation supports the notion that the release of gonadal hormones at pre- and postnatal developmental stages already has a significant influence on HPA axis (Goel et al., 2014), but to the best of our knowledge, the specific roles of male vs. female hormone on this dimorphism in young pups are yet to be addressed. Regardless, the present data clearly show that EPO overexpression in the brain effectively attenuates the NMS-induced CORT release. This is in line with *in vitro* studies indicating that EPO attenuates the ability of hypothalamic tissue extract to secrete CHR in respond to a chemical stimulus (K^+^ or veratridine; Tringali et al., 2007). This is an important observation because it clearly demonstrates EPO’s ability to modulate the HPA axis in the intact organism, even during early life.

### Early Life Stress and Disruption of the Neural Circuits That Regulate Breathing

Ventilatory measurements performed in sleeping wild-type mice show that NMS roughly doubled the occurrence of apneas during sleep, but only in males. This result is consistent with previous results obtained in rats showing that in male rats, NMS augments the respiratory instability during sleep but unlike here, females were not tested (Kinkead et al., 2009). This observation is interesting but without additional measurements, the physiological relevance of these apneas remains uncertain. The recurrent drops in arterial O_2_ resulting from apneas contribute to significant comorbidities in SA patients including hypertension (Lesske et al., 1997). In rats, the fact that NMS males (but not females) are hypertensive suggests that respiratory instabilities affect other systems. However, we do not know if apneic events are associated with significant drops in O_2_ and if they are important to the emergence of hypertension. The fact that non-sedated rodents do not tolerate pulse oximetry probes has prevented us from addressing this question. Regardless, our demonstration that in male mice NMS augments both types of apneas (spontaneous and post-sigh) concomitantly augmenting the HcVR indicates that NMS has multiple effects on the neural control of breathing in mice. Excessive chemoreflexes contribute to sleep disordered breathing in humans (Dempsey et al., 2010; White and Younes, 2012; Eckert, 2018), and the positive relationship between HcVR and apnea frequency in wild-type males is in line with phenotypic traits of many SA patients. In fact, an augmented responsiveness to CO_2_ (also known as increased loop gain) favors respiratory instability during sleep (White, 2005) and this condition contributes to the generation of central apneas in patients with heart failure (Javaheri and Dempsey, 2013; Guyenet and Bayliss, 2015). Furthermore, patients with a mixed pattern of both central and obstructive apneas have an exaggerated ventilatory response to hypercapnia (Yuan et al., 2012).

Considering that increased HVR can also contribute to respiratory instability during sleep (Soliz et al., 2016), the data from wild-type males showing that the magnitude of the HVR is inversely related to the apnea frequency were unexpected. One possible explanation would be that in mice, CO_2_-related respiratory drive is the main determinant of apneic event. Our data showing that NMS had marginal effects (if any) on the HVR in this species support this interpretation. These results differ from those reported in rats in which NMS augmented the HVR of males by ~30% while reducing that of females (Genest et al., 2004; Fournier et al., 2015). In rats, NMS augments the HcVR of females (not males), whereas as here, the augmentation was observed in wild-type mice of both sexes. Interestingly, repeated cross fostering (another form of early life stress) also augments the HcVR at adulthood equally in both sexes (D’Amato et al., 2011). Such divergences between rodent species add to the growing evidence indicating that the physiological responses of rats and mice differ substantially, especially when acute, chronic, or intermittent hypoxic challenges are involved (Lumbroso and Joseph, 2009; Jochmans-Lemoine et al., 2016, 2018). Despite some distinctions between the two animal models, the sum of the data indicates that (1) the consequences of NMS on respiratory control persist well into adulthood and (2) NMS-related respiratory disturbance (as indicated by the apnea frequency during sleep) is greater in males than females.

Recent advances in physiology have raised our awareness of the importance of sex-hormones in health and disease, including respiratory control disorders, such as sleep apnea (Kinkead et al., 2021). In rats, ovarian hormones protect females against the deleterious consequences of systemic (intermittent hypoxia) and non-systemic (NMS) stress on respiratory control dysfunction (Fournier et al., 2015; Laouafa et al., 2018, 2019; Joseph et al., 2020). Addressing the impacts of NMS in mice following loss of ovarian function would be valuable to this concept and help develop the relevance of mice as an animal model in sex-based research.

### EPO and the Regulation of Stress, and Respiratory Control

EPO continues to emerge as a promising neuroprotective factor to prevent hypoxic–ischemic injuries at adult (Brines and Cerami, 2005) and neonatal ages (McPherson and Juul, 2008; Rangarajan and Juul, 2014; Juul et al., 2015; Juul and Pet, 2015). Moreover, EPO has also great potential for the treatment of stress-related neurological disorders, including mood disorders. Studies conducted in healthy and depressed individuals showed that, similar to the effects seen with conventional antidepressants, EPO reduces the neurocognitive processing of negative emotional information while concurrently improving cognitive function (Miskowiak et al., 2008, 2010). More recently, a meta-analysis determined that EPO alleviates the cognitive deficits associated with bipolar disorder, major depression, and schizophrenia without producing a significant unfavorable impact (Li et al., 2018). These beneficial effects of EPO are consistent with EPO’s ability to regulate the activity of HPA axis (Tringali et al., 2007; Dey and Noguchi, 2017; Dey et al., 2020).

Concerning respiratory control, our laboratory demonstrated that EPO is a potent and sex-specific stimulator of the neural respiratory network in adulthood and postnatal life (Ballot et al., 2015). “*Ex-vivo*” studies using a brainstem-spinal cord preparation from newborn rodents show that EPO stimulates respiratory rhythm and prevents hypoxia-induced respiratory depression (Khemiri et al., 2012). *In vivo* studies show that in male (but not female) mice, the neonatal administration of EPO reduces apneic events on postnatal days 7, 15, and 21 (Iturri et al., 2016). Proof-of-concept experiments show that the administration of an EPO antagonist (soluble EPO – sEPO) leads to respiratory depression in normoxia, and to asphyxia and increased mortality in hypoxia (Ballot et al., 2015). Finally, by using Tg21 mice, we showed that EPO exerts a protective effect similar to that of caffeine against apneas induced by neonatal intermittent hypoxia (Laouafa et al., 2019), and adult Tg21 male mice are protected against intermittent hypoxia-induced cardiorespiratory dysfunction and oxidative stress (Elliot-Portal et al., 2018). The sum of these findings leads us to investigate the impact of NMS on Tg21 mice. In keeping with the previous finding, our results demonstrate that EPO overexpression limited the NMS-induced increase in CORT in male mice and prevented the NMS-induced increase of apnea at adult ages. Experimental manipulation of CORT levels in newborn pups and in adult mice (both wild-type and Tg21 mice) would help determine the period during which EPO most effectively protects respiratory control from the deleterious impacts of NMS.

## Conclusion and Significance

Overall, the results reported here using wild-type mice are in line with those obtained in rats showing that NMS has persistent and sex-specific effects on respiratory control. This study therefore brings valuable support to the notion that a non-systemic stress, early in life, such as NMS, is sufficient to alter this vital homeostatic function. The physiological phenotype of rodents subjected to NMS shares many features reported in humans suffering from sleep apnea, including increased respiratory instability and apneas during sleep, abnormal chemoreflexes, and a remarkable sexual dimorphism (Tenorio-Lopes and Kinkead, 2021). While similar results have been obtained using a systemic stress, such as intermittent hypoxia (IH), we must keep in mind that IH is a consequence, not a cause of SA. Although subtle, this distinction is important if we aspire to understand the origins of the disease and develop related treatment. In that regard, continuous positive airway pressure (CPAP) remains the main treatment option for SA. Although quite effective in most patients, CPAP alleviates the symptoms of SA. The results reported here provide a novel approach to the problem as our data convincingly show that attenuating HPA axis response to challenging conditions can prevent respiratory disturbance during sleep. EPO-related therapies therefore appear as a novel and promising therapy for SA patients.

## Data Availability Statement

The original contributions presented in the study are included in the article/supplementary material, further inquiries can be directed to the corresponding author.

## Ethics Statement

The animal study was reviewed and approved by Animal Protection Committee of Laval University, Québec, Canada.

## Author Contributions

EE-P, CA-R, SL, and RT performed the experiments and contributed to data analysis and manuscript writing. RK and JS contributed to data analysis and manuscript writing. All authors contributed to the article and approved the submitted version.

## Funding

JS is funded by the Canadian Institutes of Health Research (CIHR). Catalyst Grant: Sex as a Variable in Biomedical Research (SVB-158607). RK is supported by a project grant from CIHR (PJT-173396). The authors have no financial/nonfinancial arrangements or connections that are pertinent to the submitted manuscript. CA-R receives Ph.D scholarships from the “Réseau de Santé Respiratoire du Québec” and the “Fonds de Recherche du Québec-Santé” (292950).

## Conflict of Interest

The authors declare that the research was conducted in the absence of any commercial or financial relationships that could be construed as a potential conflict of interest.

## Publisher’s Note

All claims expressed in this article are solely those of the authors and do not necessarily represent those of their affiliated organizations, or those of the publisher, the editors and the reviewers. Any product that may be evaluated in this article, or claim that may be made by its manufacturer, is not guaranteed or endorsed by the publisher.
